# Molecular and Genetic Characterization of HIV-1 Tat Exon-1 Gene from Cameroon Shows Conserved Tat HLA-Binding Epitopes: Functional Implications

**DOI:** 10.3390/v8070196

**Published:** 2016-07-18

**Authors:** Georges Teto, Julius Y. Fonsah, Claude T. Tagny, Dora Mbanya, Emilienne Nchindap, Leopoldine Kenmogne, Joseph Fokam, Dora M. Njamnshi, Charles Kouanfack, Alfred K. Njamnshi, Georgette D. Kanmogne

**Affiliations:** 1 Department of Pharmacology and Experimental Neuroscience, College of Medicine, University of Nebraska Medical Center, Omaha, NE 68198-5800, USA; georges.teto@unmc.edu; 2 Faculty of Medicine and Biomedical Sciences, University of Yaoundé I, 1364 Yaoundé, Cameroon; yundze.fonsah@gmail.com (J.Y.F.); tayouclaude@yahoo.fr (C.T.T.); dmbanya1@yahoo.co.uk (D.M.); josephfokam@gmail.com (J.F.); charleskouanfack@yahoo.fr (C.K.); aknjamnshi@yahoo.co.uk (A.K.N.); 3 Department of Neurology, Yaoundé Central Hospital, 25625 Yaoundé, Cameroon; 4 Yaoundé University Teaching Hospital, 8046 Yaoundé, Cameroon; eminchindap4@yahoo.com (E.N.); l.kemmogne@yahoo.com (L.K.); 5 Sequencing and Bioinformatics Unit, Chantal BIYA International Reference Centre for Research on HIV/AIDS Prevention and Management, 3077 Yaoundé, Cameroon; 6 HIV-Day Care Service, Yaoundé Central Hospital, 87, Yaoundé, Cameroon; dmnjamnshi@yahoo.co.uk

**Keywords:** Cameroon, Tat exon-1, HIV-1 genetic diversity, *N*-myristoylation, amidation, casein kinase-2, phosphorylation, HLA binding sites

## Abstract

HIV-1 Tat plays a critical role in viral transactivation. Subtype-B Tat has potential use as a therapeutic vaccine. However, viral genetic diversity and population genetics would significantly impact the efficacy of such a vaccine. Over 70% of the 37-million HIV-infected individuals are in sub-Saharan Africa (SSA) and harbor non-subtype-B HIV-1. Using specimens from 100 HIV-infected Cameroonians, we analyzed the sequences of HIV-1 Tat exon-1, its functional domains, post-translational modifications (PTMs), and human leukocyte antigens (HLA)-binding epitopes. Molecular phylogeny revealed a high genetic diversity with nine subtypes, CRF22_01A1/CRF01_AE, and negative selection in all subtypes. Amino acid mutations in Tat functional domains included N24K (44%), N29K (58%), and N40K (30%) in CRF02_AG, and N24K in all G subtypes. Motifs and phosphorylation analyses showed conserved amidation, *N*-myristoylation, casein kinase-2 (CK2), serine and threonine phosphorylation sites. Analysis of HLA allelic frequencies showed that epitopes for HLAs A*0205, B*5301, Cw*0401, Cw*0602, and Cw*0702 were conserved in 58%–100% of samples, with B*5301 epitopes having binding affinity scores > 100 in all subtypes. This is the first report of *N*-myristoylation, amidation, and CK2 sites in Tat; these PTMs and mutations could affect Tat function. HLA epitopes identified could be useful for designing Tat-based vaccines for highly diverse HIV-1 populations, as in SSA.

## 1. Introduction

About 37 million individuals worldwide are living with HIV/AIDS and HIV-1 accounts for over 95% of all infections [1,2]. HIV-1 includes four groups: M (major), O (outlier), N (non-M non-O), and P [3,4]. HIV-1 group M accounts for the vast majority of infection globally and includes nine pure subtypes (A–D, F–H, J and K), sub-subtypes (A1 and A2, and F1 and F2), about 70 circulating recombinant forms (CRFs) and several unique (unclassified) recombinant forms (URFs) [3,5]. This high genetic variability is due to mutations and high rates of intra- and inter-molecular recombinations that occur within infected hosts, due to lack of DNA proofreading activity of the reverse transcriptase enzyme and pharmacological selective pressure [3,6,7,8].

The host genetics, ethnicity and immune response also drive HIV genetic changes and play a major role in the control of viral replication, mutations, immune response, and disease progression. In fact, it has been demonstrated that some human leukocyte antigen (HLA) alleles exert selective pressure on viral peptides, and this results in mutations that enable HIV to escape the immune system and adapt to the infected host [9,10,11]. Cytotoxic T-lymphocytes (CTL) target specific viral epitopes for the immune response and HLA alleles determine that response [11,12]. Therefore, viral escape mutations, genetic and antigenic variations have significant effects on CTL epitopes and represent major impediments to an effective immune response to any vaccine [10,13,14]. Furthermore, the HLA system is very polymorphic and factors such as geographic regions and ethnicity influence the presence of specific HLA alleles within a population [15,16]. This high polymorphism and population differences in HLA would influence the immune response to a pathogen, and there is evidence that some alleles of HLA class-I are associated with specific HIV-1 subtypes [17,18], and that some HLA genotypes such as HLA-B*5802 are associated with high viral loads and faster progression to AIDS [19,20], while others such as B*2705 and B*5701 are associated with stronger immune control and slower disease progression [21,22,23].

The HIV-1 Tat is a transcriptional regulator that is essential for viral promoter transcription, viral replication, and immune response [24,25]. Tat is expressed early in the HIV life cycle and both Tat proteins and antibodies are present in the serum of infected humans [26,27]. Since high levels of anti-Tat antibodies in HIV-infected individuals are associated with better CTL response, asymptomatic infection and slower disease progression [26,27,28,29], Tat has been considered as candidate for prophylactic and therapeutic HIV vaccine [30,31,32,33]. Animal studies [34,35] and clinical trials [30,31,32,33,36] with HIV-1 subtype-B Tat immunogens have shown that they induced Th1 and Th2 immune response and improved immune function.

Since the year 2000, over 25 million people have died from HIV/AIDS, most of them being in sub-Saharan Africa (SSA) [1,2], and many more have died since the start of this pandemic over the last three decades. With continued viral transmission and infection of new individuals, effective vaccines are needed to curb this pandemic. Although a subtype-B Tat-based vaccine could be effective in subjects infected with HIV-1 subtype-B, which is the predominant clade in Western countries, only about 12% of individuals are infected with this subtype [1,2]. Most importantly, current epidemiological data show that 25.8 million (70%) of the 37 million individuals currently living with HIV/AIDS are in SSA, are infected with non-B subtypes [1,2], and Tat vaccines based on subtype-B may not be effective in those subjects. Better knowledge of Tat genetic diversity in SSA and associated HLA epitopes in that population is critical for understanding viral evolution and designing effective Tat-based vaccines or immunotherapy for that population. There has been no previous study of HIV Tat in Cameroon, a country with generalized HIV epidemiology. In the current study, using samples from HIV-infected Cameroonians, we analyzed the structure and sequences of HIV-1 Tat exon-1, its functional domains, post-translational modifications (PTMs), and HLA-binding epitopes.

## 2. Materials and Methods

### 2.1. Study Design, Population, and Ethical Consideration

This was a cross-sectional analysis on plasma samples obtained from 100 HIV-1 infected individuals in Yaoundé, Cameroon, between 2008 and 2010. These samples were collected as part of an ongoing project aimed at analyzing the influence of HIV genetic diversity on viral neuropathogenesis in Cameroon. This study was performed in accordance with guidelines of the Helsinki Declaration and was approved by the Cameroon National Ethics Committee (National Ethical Clearance #146/CNE/SE/2012, approved on 13 June 2006 and renewed on 2 May 2012), as well as the Institutional Review Board of the University of Nebraska Medical Center (IRB# 307-06-FB, approved on 26 March 2007). Written informed consent was obtained from all participants and data were processed using unique identifiers to ensure confidentiality.

### 2.2. RNA Extraction, cDNA Synthesis and Polymerase Chain Reaction

Viral RNA was extracted from plasma samples using the QIAmp viral RNA Mini kit (Qiagen Inc., Valencia, CA, USA) according to the manufacturer’s protocol; 165 to 600 ng RNA were reverse transcribed and amplified using a nested PCR with SuperScript One-Step RT-PCR reverse transcriptase and Platinum Taq DNA polymerase (Life Technologies, Carlsbad, CA, USA), according to the manufacturer’s instructions. The 216 nucleotides Tat exon-1 was amplified in a 50 μL reaction volume containing 7.5 pmoles of each of the following forward (5′-GGATACYTGGGMAGGRGTTG-3′; 5711–5730 bp of HXB2) and reverse (5′-CATTKCCACTRTCTTCTCTC-3′; 6227–6207 bp of HXB2) primers [37]. RT-PCR was performed using the following conditions: 50 °C, 30 min; 94 °C, 2 min; 40 cycles of 94 °C, 15 s; 50 °C, 30 s; 72 °C, 1 min; and a final extension step at 72 °C, 5 min. Five microliters of each RT-PCR reaction product was used in a second/nested PCR, in a 50 μL reaction volume containing 7.5 pmoles each of the forward (5′-CAGAATTGGGTGYCAACATAG-3′; 5775–5795 bp of HXB2); and reverse (5′-CTATRGTCCACACAAYTAYKGC-3′; 6137–6116 bp of HXB2) primers, under the following conditions: 94 °C, 2 min; 40 cycles of 94 °C, 15 s; 50 °C, 30 s; 72 °C, 1 min; and a final extension step at 72 °C, 5 min. Amplicons were detected by electrophoresis on a 1% agarose gel and visualized by ethidium bromide staining under ultraviolet light.

### 2.3. DNA Sequencing and Phylogenetic Analysis

Nucleotide sequences were obtained by direct sequencing of the PCR products. Briefly, amplicons were purified using Amicon Microcon Ultra pure kit (Centrifugal Filters Devices, Millipore, Billerica, MA, USA) according to the manufacturers’ instructions. DNA sequencing was performed at the University of Nebraska Medical Center High-Throughput DNA Sequencing and Genotyping Core Facility, using a 20 μL reaction mix containing 20 ng of the purified PCR product, nested primers (12.8 pmoles forward primer or 12.8 pmoles reverse primer), and the Big-Dye chemistry method (Perkin-Elmer, Austin, TX, USA). Capillary electrophoresis was performed using an Applied Biosystems 3730 DNA sequencer (Applied Biosystems, Tokyo, Japan), and sequences were loaded and assembled into Pregap4 v.1.5 software to generate contigs [38]. Nucleotide sequences were aligned with subtype/CRFs reference sequences from the Los Alamos National Laboratory (LANL) database using the CLUSTAL.W integrated into Bioedit.7.2.5 software [39]. The phylogenetic tree was constructed by the neighbor-joining and Kimura’s two-parameter methods [40] using the MEGA.v.5 software [41]. The reliability of the branching orders was determined using 70% bootstrap robustness for subtype assignation [42,43].

### 2.4. Building of Consensus Sequences

Three to twenty Tat exon-1 sequences belonging to the same subtype were selected using BioEdit.7.2.5 [44] and the LANL HIV sequence database Consensus Maker tool [45] and aligned as fasta format in the CLUSTAL.W program to obtain a consensus sequence for each subtype. At each position, nucleotides were compared and the most frequent (50% minimal threshold) were considered in the consensus. Nucleotides with a frequency below the 50% threshold were considered missing, and gaps were treated as a fifth residue [45]. For each subtype, the validity and consistency of the selected consensus sequence was further verified by alignment with other HIV-1 Tat sequences in the database.

### 2.5. Analysis of Recombination Events

Query sequences, consensus sequences, and reference sequences were first aligned and gaps were stripped prior to subtyping analysis. Subtyping and recombination events were verified using the NCBI genotyping tool for retroviruses [46]. To ensure data accuracy, subtyping and recombination events were further confirmed using four different statistical and bioinformatics tools: the SplitsTree.4.13.1, COMET, SCUEAL, and the recombinant identification program of the HIV sequence databases. The bootscan analysis was performed using consensus CRF01_AE, CRF22_01A1, A2, A, G, C with gene specific window size and step size in Simplot v.3.5.1 to predict breakpoints within the strains [47]. Reference sequences (CRF02_AG, A1, A2, CRF09_cpx, CRF22_01A1, CRF18_cpx, CRF 19_cpx, U (unclassified), CRF13_cpx, CRF37_cpx, CRF11_cpx, CRF06_cpx, F1, F2, CRF36_cpx, CRF43_02G, CRF01_AE, CRF25_cpx; HIV-1 groups N, O, P and SIVcpz) were selected from the NCBI genotype tool for retroviruses database [46]. To determine the recombination events, gene specific window size was set at 80 bp and step size at 20 bp.

### 2.6. Identification of Mutations

Tat exon-1 nucleotide sequences were translated into amino acid (aa) sequences using the BioEdit.7.2.5 software [44]. For all viral strains, multiple sequence alignments with their corresponding consensus sequences were made using Clustal.W integrated into Bioedit.7.2.5 [39], and sequences analyzed using the LANL VESPA program [48]. This program displayed a table containing the mutations detected in the Cameroon HIV isolates, compared to corresponding consensus sequences, as well as the position of each mutation. We then analyzed the allelic frequencies of each mutation in all samples, as well as the extent of aa sequence conservation in each sample.

### 2.7. Identification of Motifs and Phosphorylation Sites

The sequence of each sample and its corresponding consensus sequence were translated into aa and analyzed using the motif scan [49,50] and NetPhos.2.0 [51] programs, to identify the motifs and phosphorylation sites in each sample (in comparison its corresponding consensus sequence), and detect the presence of unknown motifs, their positions, and match scores. The NetPhos.2.0 program was also used to identify phosphorylated aa residues, with a threshold score set at 0.500 (range 0 to 1) for prediction of phosphorylation sites.

### 2.8. Non-Synonymous/Synonymous Substitution Ratios (dn/ds)

The SNAP.2.1.1 program [52] was employed to determine the accumulation rate of non-synonymous base substitutions per potential non-synonymous site (dn) relative to the accumulation rate of synonymous base substitutions per potential synonymous site (ds). For each query sequence, the SNAP.2.1.1 program was used to calculate codon specific dn/ds ratios, compared to the corresponding subtype consensus sequence; and to determine the evolutionary pattern of codons and Tat regions under positive (dn/ds > 1) or negative (dn/ds < 1) selection. The average dn/ds values for all samples in each subtype were used for analyses.

### 2.9. Determination of HLA-Binding Peptide Motifs

Only HLAs previously shown to occur at high frequency in the Cameroonian population (including HLA-A*0201, HLA-A*0205, HLA-B*5301, HLA-B*5801, HLA-Cw*0401, HLA-Cw*0602, HLA-Cw*0702) [53,54,55] were considered in this study. The Propred-I prediction program and HTLM-II display mode [56] were used to identify promiscuous HLA-I binding sites. Because 4% is the cut-off affinity score considered sensitive and specific by the Propred-I software and HTLM-II display mode [56], we considered only epitopes with a minimum of 4% binding affinity scores. Only subtypes that included at least 5% of the Cameroon HIV-1 isolates were considered in the analysis. However, we also performed additional analyses using a clade-B consensus sequence and the clade-B Tat vaccine sequence (GenBank accession # AAA44199.1).

### 2.10. Statistical Analyses

Data were analyzed by *t*-test (two-tailed) for two-group comparisons using GraphPad Prism 5.0b. (GraphPad Software, La Jolla, CA, USA). The threshold of significance was 0.05.

## 3. Results

### 3.1. Demographic and Clinical Characteristics of Study Subjects

We analyzed plasma samples obtained between 2008 and 2010 from 100 HIV-infected Cameroonians in Yaoundé; 34 samples were from individuals with undetectable viremia and we could not amplify HIV-1 Tat from those subjects. Sixty-six samples were from individuals with detectable viremia, and we successfully amplified and sequenced HIV-1 Tat exon-1 in 60 of those samples, 53 of which were antiretroviral therapy-naïve. Subjects’ demographics and clinical characteristics are summarized in Table 1. Tat exon-1 nucleotide sequences for all 60 new clinical HIV-1 isolates analyzed in this study are available in the NCBI database; GenBank accession numbers KX360666 to KX360725.

### 3.2. Phylogenetic Analysis Shows High Genetic Diversity of Tat Exon-1 in Cameroon

**Figure 1:**
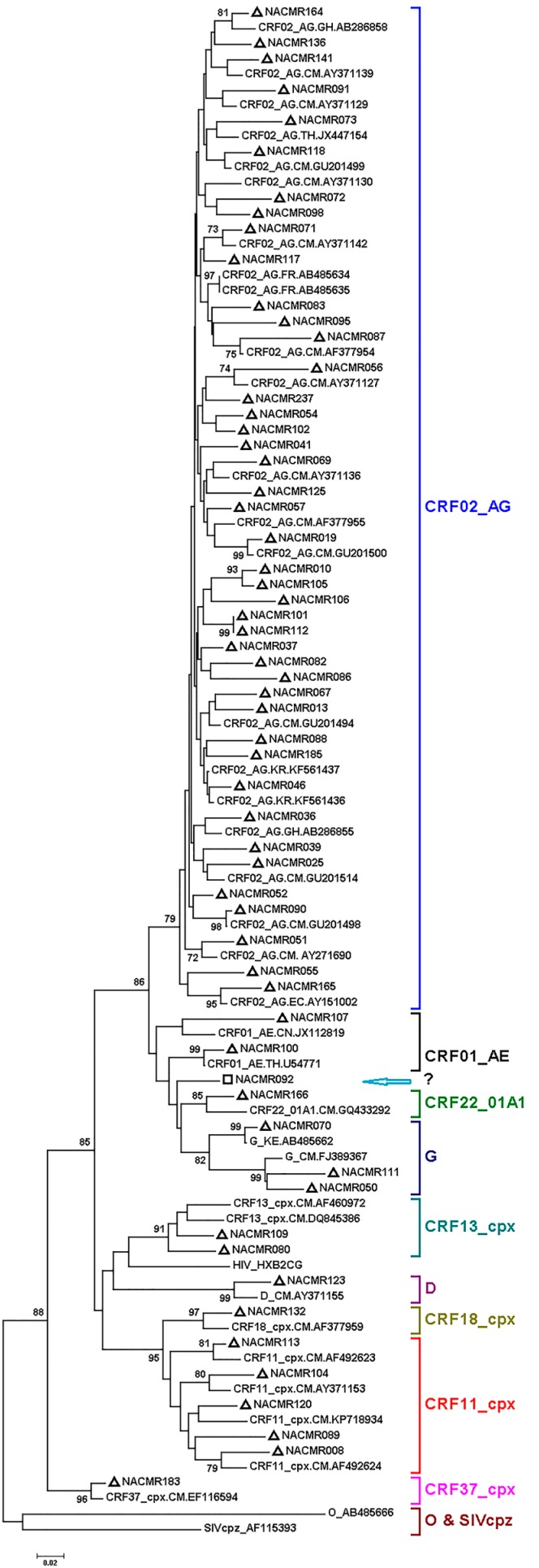
Phylogenetic analysis of Cameroon HIV-1 Tat sequences. Tat exon-1 nucleotide sequences of 60 clinical HIV-1 isolates from Cameroon (NACMR IDs) were aligned using Clustal.W, and phylogenetic analysis performed using the neighbor-joining method and MEGA.5 software as described in the Methods Section. The reference sequences were from the Los Alamos database, and included HIV-1 isolates from eight countries (Cameroon, Ghana, Kenya, France, China, Thailand, Korea, and Ecuador); some references have been omitted to enable better visualization of the new Cameroon sequences (marked by “**∆**”). The Bootstrap value of 1000 replicates of at least 70% was used to determine the HIV-1 subtype. Subject NACMR092 was infected with recombinant HIV-1 CRF01_AE/CRF22_01A1 (“**□**”, blue arrow). The scale bar represents 2% genetic distance.

**Figure 2:**
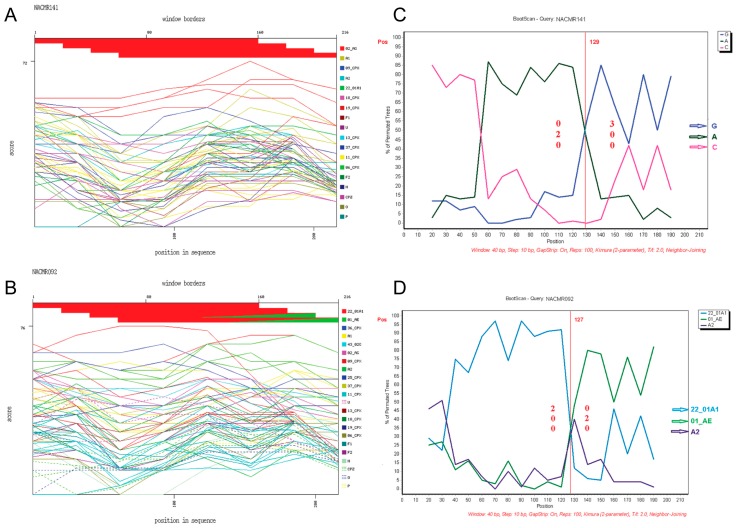
Recombination events identified and breakpoint prediction. Data show representative Tat exon-1 sequences from: a subject infected with HIV-1 CRF02_AG (NACMR141) (**A**,**C**); and a subject infected with CRF01_AE/CRF22_01A1 (NACMR092) (**B**,**D**). These clinical viral strains were genotyped using the NCBI retroviruses genotypes tool and reference subtypes (A1, A2, F1, F2, CRF02_AG, 43_02G, 01_AE, 22_01A1, 06_cpx, 09_cpx, 11_cpx, 13_cpx, 18_cpx, 19_cpx, 25_cpx, 36_cpx, 37_cpx, U (unclassified), HIV-1 groups N, O, P, and SIVcpz). For panels **A** and **B**, the red line represents CRF02_AG (**A**) or CRF22_01A1 (**B**) subtypes; and the green line represents CRF01_AE (**B**). For breakpoint prediction, bootscan analysis was performed using consensus sequences for subtypes G, A, C (panel **C**, blue, green and pink colors, respectively), or CRF22_01A1, CRF01_AE, and A2 (panel **D**, blue, green and purple colors, respectively). For all panels, the X-axis represents the percentage of sequence similarity to the corresponding subtype and the Y-axis represents the aa position of the sample sequenced.

Phylogenetic analysis of Tat exon-1 identified ten HIV-1 subtypes, with 43 subjects (71.66%) harboring CRF02_AG and 17 subjects (28.33%) with non-CRF02_AG subtypes (5 (8.33%) CRF11_cpx, 3 (5%) subtype G, 2 (3.33%) CRF01_AE, 2 (3.33%) CRF13_cpx, and 1 (1.66%) each for CRF37_cpx, CRF22_01A1, CRF18_cpx, subtype D, and CRF22_01A1/CRF01_AE) (Figure 1). The non-CRF02_AG strains identified included a URF, CRF22_01A1/CRF01_AE. Recombination analyses of this isolate, as well as a representative CRF02_AG isolate, further confirmed the identity of both the representative CRF02_AG isolate (Figure 2A) and the URF CRF22_01A1/CRF01_AE (Figure 2B). CRF22_01A1/CRF01_AE recombination was also confirmed by breakpoint analyses showing that this URF aligned with the N-terminal region of HIV-1 CRF22_01A1 and the C-terminal region of HIV-1 CRF01_AE. These findings were also supported by bootscan analyses showing with 84% and 92% confidence a recombination breakpoint occurring at the 120th nucleotide in CRF02_AG (Figure 2C) and CRF22_01A1/CRF01_AE (Figure 2D), respectively. Informative site analyses also showed that for CRF22_01A1/CRF01_AE recombinant, the N-terminal moiety consisted of 2, 0, and 0 value for subtypes CRF22_01A1, CRF01_AE, and A2 respectively, while the C-terminal moiety consisted of 0, 2, and 0 value for CRF22_01A1, CRF01_AE, and A2 respectively (Figure 2D), confirming that CRF22_01A1 is in the N-terminal and CRF01_AE in the C-terminal region of this URF. Similarly, informative site analyses of CRF02_AG showed that its N-terminal moiety consisted of 0, 2, and 0 value for subtypes G, A, and C, respectively, and its C-terminal moiety consisted of 3, 0, and 0 value for G, A, and C, respectively (Figure 2C).

### 3.3. Mutations in Cameroon HIV-1 Tat Functional Domains

**Figure 3:**
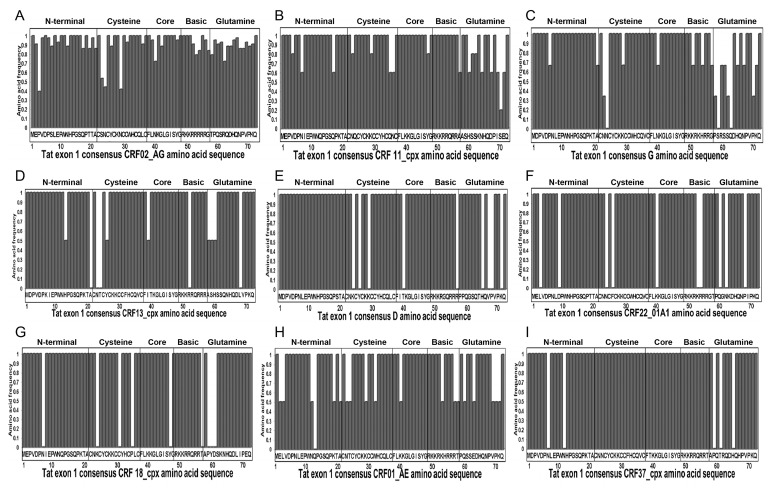
Tat exon-1 amino acid (aa) sequences of Cameroon clinical isolates. Data show Cameroon HIV-1 strains Tat exon-1 aa signature patterns for: HIV-1 CRF02_AG (**A**); CRF11_cpx (**B**); subtype G (**C**); CRF13_cpx (**D**); subtype D (**E**); CRF22_01A1 (**F**); CRF18_cpx (**G**); CRF01_AE (**H**); and CRF37_cpx (**I**); compared to consensus sequences (X-axis). The Y-axis represents the aa frequency in samples in each subtype. The Tat functional domains are shown: N-terminal (aa 1–21), Cysteine-rich (aa 22–37), Core (aa 38–48), Basic/TAR-binding (aa 49–57), and Glutamine-rich (aa 58–71) regions.

Analysis of aa sequences showed mutations in Tat functional domains of Cameroon viral isolates, compared to consensus sequences (Figure 3 and Table 2). Mutations identified in the Tat cysteine-rich region included N29K and N36I in CRF02_AG and CRF11_cpx viral isolates, respectively (Figure 3A,B); N23T, N24K, and K29T in subtype-G isolates (Figure 3C); N23T and T24K in CRF13_cpx (Figure 3D); K24S, Y26H, and K29H in subtype-D (Figure 3E); N24K and F26W in CRF22_01A1 (Figure 3F); K24Q, C31S, and P35Q in CRF18_cpx (Figure 3G); and N23S and K29R in CRF01_AE isolates (Figure 3H). No mutation was observed in the cysteine-rich region of CRF37_cpx (Figure 3I).

Mutations identified in the Core region included I39T in CRF13_cpx (Figure 3D); K40N in CRF22_01A1 (Figure 3F) and Y47H in CRF18_cpx isolates (Figure 3G). No mutation was observed in the Core region of CRF37_cpx, CRF11_cpx, CRF01_AE, CRF02_AG, subtypes G, and D isolates (Figure 3). Mutations in the Basic (TAR-binding) region included A57T in CRF11_cpx (Figure 3B); R52W in CRF13_cpx (Figure 3D); K53R and R54H in CRF22_01A1 (Figure 3F); T57A in CRF18_cpx (Figure 3G), K53R in CRF01_AE (Figure 3H); and R57G in subtype-D isolates (Figure 3E). No mutation was observed in the Basic region of CRF02_AG or CRF37_cpx (Figure 3A,I). Mutations in the glutamine (Q)-rich region included S70P in CRF11_cpx (Figure 3B); P58T, S59P, S62N, Q63K, and P70Q in subtype-G (Figure 3C); A58T, S59T, H60P, and L68P in CRF13_cpx (Figure 3D); P59A, T61S, and H65N in CRF37_cpx (Figure 3I); H65N, V67D, P68S, and K71N in subtype-D (Figure 3E); Q60H and P68L in CRF22_01A1 (Figure 3F); P59S, Y60H, and D61S in CRF18_cpx (Figure 3G); E63K, P70S, and K71Q in CRF01_AE isolates (Figure 3H). Overall, the mutations identified were highly prevalent in the samples analyzed. All mutations identified in CRF37_cpx, CRF22_01A1, CRF18_cpx, and subtype-D isolates were present in 100% of samples analyzed (Table 2), and mutations identified in the CRF02_AG, CRF11_cpx, CRF13_cpx, CRF01_AE, and subtype-G isolates were present in 40%–100% of samples analyzed (Table 2), with the exception of K29T mutation in subtype-G cysteine-rich region that was present in only 33.3% of samples analyzed (Figure 3 and Table 2).

### 3.4. Mutations in Cameroon HIV-1 Tat Functional Domains are Associated with Potential Post-Translational Modifications (PTMs)

**Figure 4:**
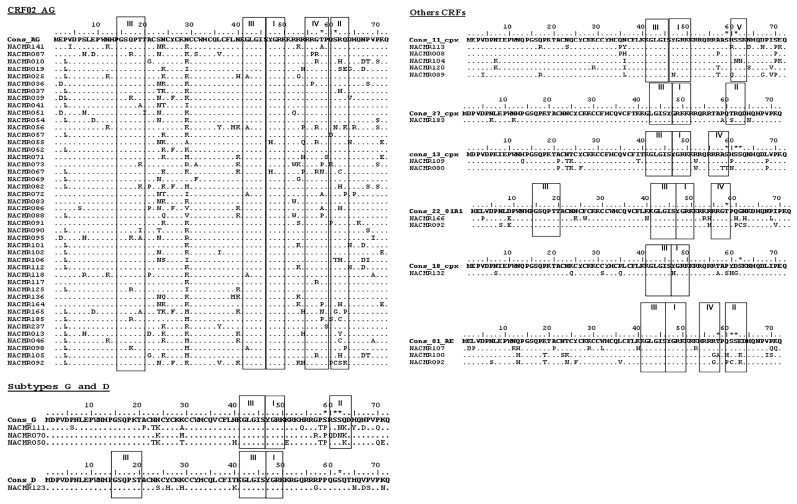
Motifs and post-translational modification (PTM) sites in Tat exon-1 of Cameroon clinical HIV-1 isolates. The Motif scan and Netphos.2.0 software were used to predict motifs and PTM sites in Cameroon Tat aa sequences as described Methods. For each subtype (CRF02_AG, G, D, CRF11_cpx, CRF37_cpx, CRF13_cpx, CRF22_01A1, CRF18_cpx, and CRF01_AE), consensus sequences are shown, as well as the IDs of Cameroon samples. Data show the presence of amidation (**I**); casein kinase-II (**II**); *N*-myristoylation (**III**); cAMP protein kinase (**IV**); and protein kinase-C (**V**) sites; as well as the predicted phosphorylation sites and phosphorylated aa residues (asterisk). Dots indicate conserved aa compared to corresponding consensus sequences.

Since mutations at specific aa residues can affect PTMs and Tat function, including viral transactivation [57,58], we analyzed the motifs and predicted PTMs in Tat functional domains of Cameroon HIV-1 isolates. Compared to consensus sequences, arginine (R) residues in the TAR-binding domain were overall conserved in Cameroon isolates (Figure 4); and in the glutamine-rich region, Q residues were conserved in 77% of CRF02_AG isolates and were mostly conserved in other subtypes (Figure 4). Compared to consensus sequences, C residues in the cysteine-rich region were conserved in 93% of samples analyzed; only three CRF02_AG isolates showed mutations at C31 (C31S/A/F), and one CRF18_cpx isolate showed a C31S mutation (Figure 4). Several CRF02_AG samples showed mutations of asparagine (N) into lysine (K) in the cysteine-rich and Core regions, including N24K, N29K, and N40K in 44%, 58%, and 30% of samples, respectively (Figure 4). All subtype-G samples also showed N24K mutation, and 42% of CRF02_AG samples had S23N substitution (Figure 4). Compared to consensus sequences, no major mutations were observed in the Tat N-terminal region of Cameroon isolates except the P3L substitution in 63% of CRF02_AG isolates (Figure 4).

Analysis showed the presence of functional protein motifs in Tat of Cameroon HIV-1 isolates. All samples showed the presence of an *N*-myristoylation domain in the Core region, and CRF02_AG, CRF22_01A1, and subtype-D isolates had an additional *N*-myristoylation domain in the N-terminal region (Figure 4, III). All samples also showed an amidation domain spanning the Core and TAR-binding regions (Figure 4, I). A cAMP protein kinase (PK) domain (PKA) spanning the TAR-binding and glutamine-rich regions was present in Tat sequences of CRF02_AG, CRF13_cpx, CRF22_01A1, and CRF01_AE viral isolates (Figure 4, IV). A casein kinase-2 (CK2) domain was present in the glutamine-rich region of CRF02_AG, subtype-G, CRF37_cpx, and CRF01_AE Tat sequences (Figure 4, II), and CRF11_cpx isolates also showed a PKC domain in the glutamine-rich region (Figure 4, V).

Analysis also showed phosphorylation site within serine residues (S59, S61, S62) in CRF02_AG, subtypes G and D, CRF11_cpx, CRF13_cpx, CRF18_cpx, and CRF01_AE Tat; and phosphorylation of threonine residues (T58) in CRF02_AG, CRF22_01A1, and CRF01_AE Tat (Figure 4). No aa phosphorylation site was detected in CRF37_cpx Tat sequences. Interestingly, serine and threonine phosphorylation occurred only in the glutamine-rich regions and serine phosphorylation occurred only in CK2 or PKC motifs, whereas threonine phosphorylation occurred only in PKA motifs (Figure 4).

### 3.5. Selection Pressure and HLA-Binding Motifs in Cameroon HIV-1 Tat Sequences

Given that the dn/ds ratio can influence viral sequence evolution, viral adaptation, and disease progression [59], we analyzed the Tat dn/ds ratios of Cameroon HIV-1 isolates. Fifty-seven of the 60 (95%) HIV-1 Tat sequences analyzed showed a purifying selection, with dn/ds ratios < 1 (Table 3). Tat sequences from three individuals infected with HIV-1 CRF02_AG had dn/ds ratios between 1.27 and 2.07, but the other 40 individuals with HIV-1 CRF02_AG had dn/ds ratios < 1 and the overall mean dn/ds ratio for all 43 CRF02_AG infected subjects was below 0.4 (Table 3).

**Figure 5:**
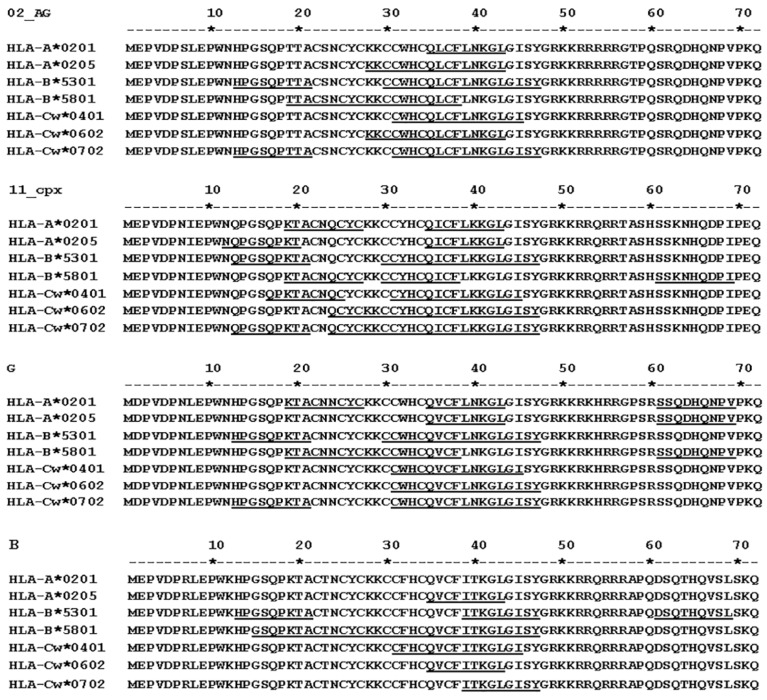
ProPred analysis of Tat exon-1 HLA binding epitopes for three HIV-1 subtypes predominant in Cameroon, and HIV-1 subtype B. HLA motifs were identified using the ProPred.1 HLAs Binding Prediction software as described in Methods. The HLA alleles and their predicted binding sequences (underline) for HIV-1 CRF02_AG, CRF11_cpx, and subtype G isolates in Cameroon are shown.

We identified and analyzed the binding affinity of HLA motifs in Tat sequences of Cameroon HIV-1 isolates, focusing on HLAs that were previously shown in Cameroon populations [53,54,55], subtypes identified in at least 5% of the samples analyzed, and subtype B. For all four subtypes analyzed (CRF02_AG, CRF11_cpx, G, and B), the HLA-B*5301 allele showed epitopes that had binding affinity scores > 100; the HLA-Cw*0401 allele had epitopes with binding affinity scores of 25 to 600; and the HLA-A*0205, HLA-Cw*0401, HLA-Cw*0602, and HLA-Cw*0702 alleles had epitopes with binding affinity scores of 4 to 25 (Table 4, Figure 5). The HLA-B*5801 allele had epitopes with binding affinity scores of ≥4 only in subtype B and CRF11_cpx (Table 4). HLA alleles and epitopes present in CRF02_AG, CRF11_cpx, and subtype G Tat sequences were conserved, respectively, in 58% to 81%, 60% to 100%, and 67% to 100% of samples analyzed (Table 4). The epitope HPGSQPKTA in subtype B HLA-B*5301 allele was also present in 66% of subtype G isolates, but none of the other subtype B epitopes identified was present in the Cameroon isolates analyzed (Table 4). Subtype B data shown (Table 4 and Figure 5) were obtained using a B consensus sequence. Additional analyses using the subtype B Tat vaccine sequence (GenBank accession # AAA44199.1) gave similar results.

## 4. Discussion

This is, to our knowledge, the first study of HIV-1 Tat sequences in Cameroon. This comprehensive molecular study showed high genetic diversity of HIV-1 Tat exon-1 among infected subjects in Cameroon, in agreement with diversity shown for HIV-1 gag, pol, env, nef genes in Cameroon [60,61,62,63,64,65]. Our analysis of Tat exon-1 sequences confirmed these findings, and showed a predominance of CRF02_AG (71.6% of samples analyzed), similar to previous studies that showed based on the analysis of env, gag, pol, and nef genes that HIV-1 CRF02_AG represented 48% to 74% of viral strains in Cameroon [60,61,62,63,64].

A previous analysis of env, gag and pol sequences in a sample from an infected individual in Bertoua (Eastern region of Cameroon) concluded that the individual was infected with a novel unique recombinant HIV-1 CRF22_01A1/CRF01_AE [66]. Of relevance, our current analysis of Tat sequences showed that an individual from Yaoundé (Central region of Cameroon) was infected with HIV-1 CRF22_01A1/CRF01_AE (NACMR092). This is the second report of infection with CRF22_01A1/CRF01_AE in the literature, and the fact that this mosaic virus has been identified in two different Cameroonians who had no apparent epidemiological link, suggests that CRF22_01A1/CRF01_AE is spreading in Cameroon. Although these two pieces of evidence were not generated from the same genes, subsequent full-length analysis would be more informative.

There have been reports of other CRF22_01A1 recombinants in Cameroon; analyses of gag, pol, and env sequences in samples from infected Cameroonians showed recombinants of CRF22_01A1 with CRF02_AG, CRF11_cpx, and clades A [66,67], confirming recombination hotspots between HIV-1 strains circulating in Cameroon. In fact, genetic recombination often occurs at hotspot regions; hotspot motifs are found at breakpoint regions and are associated with genomic instability and evolution [68,69]. Such recombination events can lead to the rearrangement of gene sequences, which in turn contribute to the wide viral heterogeneity and evolution in SSA, thus increasing viral adaptability to selective pressure. In fact, all subtypes in our study had dn/ds ratios of <1, indicating a negative selection. These data show that HIV-1 genetic diversity in Cameroon may be associated with mutations and allelic purification in the viral Tat sequences that might improve/increase viral fitness and adaption in the human host.

Amino acid substitutions identified in Cameroon Tat sequences included N24K, N29K, and N40K respectively in 44%, 58%, and 30% of CRF02_AG samples and N24K in all subtype G samples. Such mutations can have significant functional implications. Lysine-associated hydrogen bonds are important for protein stability and K residues play a significant role in HIV-1 Tat transactivation. Studies of HIV-1 subtypes B, C and CRF01_AE Tat exon-1 showed that compared to subtype B Tat, there was a significant increase in viral transactivation with CRF01_AE and subtype C Tat [70]; K residues in the cysteine-rich, Core, and TAR-binding regions played an important role in this increased Tat activity and viral transactivation, and mutations of K residues to A decreased viral transactivation by two to 20 folds [70]. Thus, it is possible that mutations resulting in increased K residues, as shown in our studies for CRF02_AG and subtypes G isolates, could result in increased LTR transactivation and viral replication in subjects infected with those subtypes.

PTMs of proteins modulate their structure and function, including their signaling and interactions with other molecules and co-factors. Thus, identifying Tat PTMs and associated motifs is critical for understanding its function in disease and future therapeutic vaccine development. In the current study, we identify five different PTMs sites in Tat exon-1 of Cameroon HIV-1 isolates. This is, to our knowledge, the first study to show the presence of amidation and *N*-myristoylation sites in Tat proteins. It is likely that amidation plays a role in Tat function, as other studies showed that amidation of neuropeptides is important for their activity, bioavailability, and biological function [71]. Myristoylation plays an important role in protein signaling and function, as the myristoyl moiety guides the protein subcellular localization, its interaction with cellular membranes and other proteins [72,73,74]. In fact, myristoylation has been shown to play a major role in HIV infection. Myristoylation of the matrix domain of HIV Gag and Gag-Pol precursor protein is necessary for Gag anchoring to the plasma membrane, viral assembly, and the formation of mature infectious viral particles [75,76]; and inhibiting myristoylation blocks the formation of competent virions [77,78]. Nef myristoylation is also required for the incorporation of virions into cells [79,80], and is associated with enhanced HIV replication and progression to AIDS [79,81]. Our data showing myristoylation of Tat in Cameroon HIV isolates suggest that *N*-myristoylation could be playing a role in Tat function, including viral transactivation, Tat cellular uptake and cytotoxicity. Significantly, HIV-1 CRF02_AG, CRF22_01A1, and subtype D isolates had two *N*-myristoylation motifs whereas other subtypes had only one *N*-myristoylation motif in the Core region. Considering the importance of *N*-myristoylation in HIV subcellular location, viral assembly, transmission and replication [75,76,77,78,79]; as well as the importance of *N*-myristoylation of HIV proteins in signaling, inflammation, and progression to AIDS [79,80,81]; the increased number of *N*-myristoylation motifs in CRF02_AG, CRF22_01A1, and subtype D isolates could suggest enhanced Tat function in those viral subtypes. This may include increased transactivation and viral replication with Tat CRF02_AG, CRF22_01A1, and subtype D compared to Tat of other HIV-1 subtypes (CRF11_cpx, CRF37_cpx, CRF13_cpx, CRF18_cpx, CRF01_AE, subtype G) that had only one *N*-myristoylation motif. Our future studies will test this hypothesis, and determine which glycine residues in the Tat *N*-myristoylation motifs (G15, G42, or G44) are myristoylated.

Other PTMs sites identified in Tat sequences included a CK2 motif in four subtypes (CRF02_AG, CRF37_cpx, CRF01_AE, and subtype G), a PKA motif in four subtypes (CRF02_AG, CRF13_cpx, CRF22_01A1, and CRF01_AE), and a PKC motif in one subtype (CRF11_cpx). CK2, PKC and PKA are all serine/threonine kinases that play major role in the phosphorylation of serine and threonine residues, cellular signaling and regulation of HIV transcription [82,83,84,85,86,87]. HIV uses cellular CK2 to phosphorylate viral proteins, and CK2 mediates the phosphorylation of Rev, Vpu, and protease at serine residues to facilitate HIV infection, syncytia formation [82,83,84,85], and disease progression in simian/human immunodeficiency virus-infected primates [88]. CK2-mediated phosphorylation of Rev serine residues influenced Vpu interaction with CD4 [89,90], and mutations in CK2 site significantly altered Vpu biological activity [91]. There has been, to our knowledge, no previous study showing CK2 domains in HIV-1 Tat. Our current study showing serine/threonine kinases phosphorylation sites in Cameroon Tat sequences suggests that CK2 modulates the signaling and biological activity of Tat during infections with HIV-1 CRF02_AG, CRF37_cpx, CRF01_AE, and subtype G by phosphorylating S61 and/or S62; that PKA modulates the signaling and function of Tat during infections with HIV-1 CRF02_AG, CRF13_cpx, CRF22_01A1, and CRF01_AE by phosphorylating T58, T59 or S59; and that PKC modulates the signaling and biological activity of Tat during infections with HIV-1 CRF11_cpx by phosphorylating S60 and S62.

High levels of anti-Tat antibodies in subjects infected with subtype B HIV-1 are associated with better CTL immune response [26,27,28,29,92], and there have been efforts to develop a subtype B Tat-based vaccine [30,31,32,33]. Given that HLA variations among different populations would influence viral evolution, genetic diversity, immune response and the efficacy of such Tat-based vaccine [20,93], we analyzed HLA motifs in Tat sequences of Cameroon HIV-1 isolates. Our data showed that epitopes for HLAs A*0205, B*5301, Cw*0401, Cw*0602, and Cw*0702 were present in 58% to 100% of all samples analyzed, with B*5301 epitopes having binding affinity scores > 100 in all subtypes analyzed. CTL target specific HIV protein epitopes for immune control and HLA alleles determine that CTL response, including recognition and binding to T-cell receptors [19,93,94]. Our data suggests that Tat-based immunogens targeting HLAs B*5301 and/or Cw*0401 epitopes could work for most Cameroonians infected with HIV-1 CRF02_AG, CRF11_cpx, and subtype G; could also work in some subtype B-infected subjects; and that multi-epitope constructs of these HLAs alleles identified could work for infected subjects in Cameroon. Our subsequent studies will further investigate the frequency and affinities of these HLA epitopes in other HIV subtypes in SSA, including subtype C that is highly prevalent in Southern and Eastern Africa. We will also analyze the relationship between HLAs B*5301 and Cw*0401 epitopes and the clinical status of HIV-infected Cameroonians; as studies of subjects infected with subtype B HIV-1 showed that some HLA alleles such as HLA B*5701 and HLA-B*27 correlate with better immune control and slower progression to AIDS [21,22,23], while other alleles such as HLA-B*5802 correlate with faster disease progression [19,20].

## 5. Conclusions

The current study is, to the best of our knowledge, the first analysis of HIV-1 Tat-exon-1 in Cameroon. Our data confirm the broad HIV genetic diversity in Cameroon with predominant CRF02_AG. Our data also show a negative selection for all subtypes and the presence of CRF22_01A1/CRF01_AE, the only second report of such recombinant in the literature. Furthermore, we showed the presence of conserved PTMs motifs in Tat functional domains, including *N*-myristoylation, amidation, and CK2 sites. This is, to our knowledge, the first study to show *N*-myristoylation, amidation, and CK2 motifs in Tat sequences, and our future studies will investigate the role of these PTMs in Tat-mediated signaling and biological function. We also showed conserved Tat HLA-binding epitopes that had high frequencies and high affinity, and this could be useful for future multi-epitope vaccine constructs for Cameroonian and SSA populations.

## Figures and Tables

**Table 1:** Demographics and laboratory characteristics of patients included in the study.

Characteristics	Male	Female	*p*-Value
N (%)	17 (28.33%)	43 (71.66%)	
Age (mean ± SD)	39.5 ± 8.67	36 ± 9.95	0.214
Age range	(23–58)	(20–56)	
Education (years; mean ± SD)	9.76 ± 3.3	9.14 ± 3.6	0.547
Education range (years)	(5–16)	(4–18)	
Mean CD4 ± SD (Cells/µL)	443.2 ± 218.9	285.5 ± 185.8	0.009
CD4 range (Cells/µL)	65–732	12–722	
CD4 IQR (Cells/µL)	250–654	122–418	
Mean viral load ± SD (Log copies/mL)	5.47 ± 5.16	5.9 ± 5.5	0.41
Viral load range (Log copies/mL)	(3.9–6.3)	(1.54–7)	
ART-naïve (N, %)	15 (79%)	38 (92.7%)	

**Table 2:** Mutations in Tat exon-1 of Cameroon HIV-1 isolates.

Subtypes	Mutations	Functional Domain	Allele Frequency
CRF02_AG	P3L	N-terminal region	0.605
	N29K	Cysteine rich	0.581
CRF11_cpx	N36I	Cysteine rich	0.400
	A57T	Arginine rich	0.400
	S70P	Glutamine rich	0.800
CRF13_cpx	H13Q	N-terminal region	0.500
	A21P	N-terminal region	1.000
	N23T	Cysteine rich	1.000
	T24K	Cysteine rich	1.000
	I39T	Core region	0.500
	R52W	Arginine rich	1.000
	A58T	Glutamine rich	0.500
	S59T	Glutamine rich	0.500
	H60P	Glutamine rich	0.500
	L68P	Glutamine rich	1.000
CRF37_cpx	N7K	N-terminal region	1.000
	N12K	N-terminal region	1.000
	P59A	Glutamine rich	1.000
	T61S	Glutamine rich	1.000
	H65N	Glutamine rich	1.000
CRF22_01A1	L3P	N-terminal region	1.000
	D9E	N-terminal region	1.000
	N24K	Cysteine rich	1.000
	F26W	Cysteine rich	1.000
	K40N	Core region	1.000
	K53R	Arginine rich	1.000
	R54H	Arginine rich	1.000
	Q60H	Glutamine rich	1.000
	N62H	Cysteine rich	1.000
	P68L	Glutamine rich	1.000
CRF18_cpx	N7S	N-terminal region	1.000
	K24Q	Cysteine rich	1.000
	C31S	Cysteine rich	1.000
	P35Q	Cysteine rich	1.000
	Y47H	Core region	1.000
	T57A	Arginine rich	1.000
	P59S	Glutamine rich	1.000
	Y60H	Glutamine rich	1.000
	D61S	Glutamine rich	1.000
CRF01_AE	L3P	N-terminal region	0.500
	Q13H	N-terminal region	1.000
	K19T	N-terminal region	0.500
CRF01_AE	A21P	N-terminal region	0.500
	N23S	Cysteine rich	0.500
	K29R	Cysteine rich	0.500
	K53R	Arginine rich	0.500
	E63K	Glutamine rich	0.500
	P70S	Glutamine rich	0.500
	K71Q	Glutamine rich	0.500
G	N23T	Cysteine rich	0.667
	N24K	Cysteine rich	1.000
	K29T	Cysteine rich	0.333
	P58T	Glutamine rich	0.667
	S59P	Glutamine rich	1.000
	S62N	Glutamine rich	0.667
	Q63K	Glutamine rich	1.000
	P70Q	Glutamine rich	0.667
D	K24S	Cysteine rich	1.000
	Y26H	Cysteine rich	1.000
	K29H	Cysteine rich	1.000
	T40K	Arginine rich	1.000
	R57G	Arginine rich	1.000
	H65N	Glutamine rich	1.000
	V67D	Glutamine rich	1.000
	P68S	Glutamine rich	1.000
	K71N	Glutamine rich	1.000

**Table 3:** Tat exon-1 subtypes dn/ds ratios.

Subtypes	N (%)	Consensus	Average dn/ds Ratios
CRF02_AG	43 (71.6)%	02_AG	0.399
CRF11_cpx	5 (8.8%)	11_cpx	0.162
G	3 (5%)	G	0.357
CRF13_cpx	2 (3.3%)	13_cpx	0.440
CRF01_AE	2 (3.3%)	01_AE	0.515
CRF18_cpx	1 (1.6%)	18_cpx	0.341
CRF22_01A1	1 (1.6%)	22_01A1	0.930
CRF37_cpx	1 (1.6%)	37_cpx	0.140
D	1 (1.6%)	D	0.930
CRF01_AE/22_01A1	1 (1.6%)	01_AE	0.536
CRF01_AE/22_01A1	1 (1.6%)	22_01A1	0.434

**Table 4:** HLA binding epitopes of Tat exon-1 sequences present in Cameroon HIV-1 isolates and subtype B.

Alleles	Epitope Sequence	Start Residue	Frequency (%)	Binding Affinity Score
**CRF02_AG**				
HLA-A*0201	QLCFLNKGL	35	58.13	21.3624
HLA-A*0205	QLCFLNKGL	35	58.13	7
HLA-B*5301	HPGSQPTTA	13	69.76	119.69
	LNKGLGISY	39	62.79	106.1
	CCWHCQLCF	30	72.09	103.98
HLA-Cw*0401	CFLNKGLGI	37	81.39	25
	QLCFLNKGL	35	58.13	4
HLA-Cw*0602	QLCFLNKGL	35	58.13	6.6
HLA-Cw*0702	LNKGLGISY	39	62.79	8
**CRF11_cpx**				
HLA-A*0205	QICFLKKGL	35	60	7
HLA-B*5301	QPGSQPKTA	13	100	118.6
	LKKGLGISY	39	80	106.1
HLA-B*5801	KTACNQCYC	19	80	6
HLA-Cw*0401	CYHCQICFL	31	60	600
	QICFLKKGL	34	60	4
HLA-Cw*0602	QICFLKKGL	35	60	6.6
	CYHCQICFL	31	60	4
HLA-Cw*0702	LKKGLGISY	39	80	8
	CYHCQICFL	31	60	4.32
**Subtype G**				
HLA-A*0205	QVCFLNKGL	35	100	14
HLA-B*5301	HPGSQPKTA	13	66.66	118.81
	LNKGLGISY	39	66.66	106.1
	CCWHCQVCF	30	100	103.98
HLA-Cw*0401	CWHCQVCFL	31	66.66	120
	QVCFLNKGL	35	100	4
HLA-Cw*0602	QVCFLNKGL	35	100	6.6
	CWHCQVCFL	31	66.66	4
HLA-Cw*0702	LNKGLGISY	39	66.66	8	
**Subtype B**				
HLA-A*0205	QVCFITKGL	35	N/A	14
HLA-B*5301	HPGSQPKTA	13	66.66	118.81
	ITKGLGISY	39	N/A	106.1
	DSQTHQVSL	61	N/A	104.84
	CCFHCQVCF	30	N/A	103.98
HLA-B*5801	ITKGLGISY	39	N/A	13.5
	KTACTNCYC	19	N/A	6
	GSQPKTACT	15	N/A	4
HLA-Cw*0401	CFHCQVCFI	31	N/A	75
	CFITKGLGI	37	N/A	25
HLA-Cw*0602	QVCFITKGL	35	N/A	6.6
HLA-Cw*0702	ITKGLGISY	39	N/A	8

N/A: not available (not found in any Cameroon Tat sequences).
